# Morphology, Phylogeny, and Pathogenicity of Pestalotioid Species on *Camellia oleifera* in China

**DOI:** 10.3390/jof7121080

**Published:** 2021-12-15

**Authors:** Lingling Li, Qin Yang, He Li

**Affiliations:** 1 Key Laboratory for Non-Wood Forest Cultivation and Conservation of the Ministry of Education, Central South University of Forestry and Technology, Changsha 410004, China; 20191200035@csuft.edu.cn; 2 Key Laboratory of National Forestry and Grassland Administration for Control of Diseases and Pests of South Plantation, Central South University of Forestry and Technology, Changsha 410004, China; 3 Hunan Provincial Key Laboratory for Control of Forest Diseases and Pests, Central South University of Forestry and Technology, Changsha 410004, China; 4 Hunan Provincial Key Laboratory for Forestry Biotechnology, Central South University of Forestry and Technology, Changsha 410004, China

**Keywords:** five new taxa, *Neopestalotiopsis*, *Pestalotiopsis*, phylogeny, taxonomy

## Abstract

Tea-oil tree (*Camellia oleifera*) is an important edible oil woody plant with a planting area of over 3,800,000 hectares in southern China. Pestalotioid fungi are associated with a wide variety of plants worldwide along with endophytes, pathogens, and saprobes. In this study, symptomatic leaves of *C. oleifera* were collected from Guangdong, Guangxi, Hainan, Hunan, and Jiangsu Provinces and pestalotioid fungi are characterized based on combined sequence data analyses of internal transcribed spacer (ITS), beta tubulin (*tub2*), and translation elongation factor 1-alpha (*tef-1α*) coupled with morphological characteristics. As a result, seven species were confirmed, of which five species are described as new viz. *N. camelliae-oleiferae*, *P. camelliae-oleiferae*, *P. hunanensis*, *P. nanjingensis*, *P.* *nanningensis*, while the other two are reported as known species, viz., *N. cubana* and *N. iberica*. Pathogenicity assays showed that all species except for *P. nanjingensis* developed brown lesions on healthy leaves and *P. camelliae-oleiferae* showed stronger virulence.

## 1. Introduction

Tea-oil tree (*Camellia oleifera* Abel.) is a unique woody edible oil species in China, mainly distributed in the Qinling-Huaihe River area. It has a long history of cultivation and utilization for more than 2300 years since ancient China [1]. Statistical data for 2014 indicated that these plantations comprise over 3,800,000 hectares and produce 518,000 tons of edible oil (State-owned Forest Farms and Nurseries Station, State Forestry Administration of China, 2016). Camellia oil, obtained from *C. oleifera* seeds, is rich in unsaturated fatty acids and unique flavors, and has become a rising high-quality edible vegetable oil in China [2]. Thus, the development of the *C. oleifera* industry is of great significance for the national economy and poverty alleviation of local farmers in China.

The expanding cultivation of *C. oleifera* over the last several decades has also attracted increasing attention from plant pathologists to infectious diseases on this crop. Anthracnose disease caused by *Colletotrichum* species is one of the foremost diseases in southern China, which can infect leaves and fruits of *C. oleifera*, causing up to a 40% fruit drop and up to 40% camellia seeds loss [3]. Several studies have focused on the diversity and the pathogenicity of fungi in this special habitat [3,4,5]. However, relatively little is known about the taxonomy, genetic diversity, and pathogenicity of pestalotioid species on *C. oleifera*.

Pestalotioid species represent a cosmopolitan group of fungi occupying diverse ecological behavior as plant pathogens, endophytes, or saprobes, and are widely distributed throughout tropical and temperate regions [6,7,8]. However, species identification in this genus remains a major challenge because of overlapping conidial measurements [6,7,9,10]. Maharachchikumbura et al. [8] segregated *Neopestalotiopsis* and *Pseudopestalotiopsis* from *Pestalotiopsis*, based on conidial pigment color, conidiophores and multi-locus phylogenetic analyses. *Neopestalotiopsis* can be easily distinguished from *Pseudopestalotiopsis* and *Pestalotiopsis* by its versicolorous median cells [8]. *Pseudopestalotiopsis* differs from *Pestalotiopsis* by having three darker median cells and knobbed apical appendages [8]. Many novel species were introduced into this group during recent years through a polyphasic approaches together with morphology [11,12,13,14,15,16,17,18,19,20,21]. This study aimed to identify the pestalotioid fungi associated with *Camellia oleifera* in China based on both morphological characters and molecular phylogeny.

## 2. Materials and Methods

### 2.1. Sample Collection and Isolation

The isolates in this study were collected from *Camellia oleifera* with irregular, brownish-grey lesions on leaves, and accounted for 25% of the surveyed leaves. Samples were obtained from the main tea-oil camellia production fields in Guangdong, Guangxi, Hainan, Hunan, and Jiangsu Provinces in 2020. Small sections (3 × 3 mm) were cut from the margins of infected tissues, and surface-sterilized in 75% ethanol for 30 s, then sterilized in 5% (*vol**/**vol*) sodium hypochlorite for 1 min, followed by three rinses with sterilized water and finally dried on sterilized filter paper. The sections were then plated onto PDA plates and incubated at 25 °C. Fungal growth was examined daily for up to 7 d. Isolates were then transferred aseptically to fresh PDA and purified by single-spore culturing. All fungal isolates were placed on PDA slants and stored at 4 °C. Specimens and isolates of the new species have been deposited in the Central South University of Forestry and Technology Culture Collection (CSUFTCC). 

### 2.2. Morphological and Cultural Characterization

Colony characteristics of cultures on potato dextrose agar (PDA) medium were recorded after 7 d incubation at 25 °C. Fungal morphology was recorded from colonies grown in the dark for 14 d at 25 °C on PDA. The morphological characteristics were examined by mounting fungal structures in clear lactic acid and 30 measurements at ×1000 magnification were determined for each isolate using a Leica compound microscope (DM 2500) with interference contrast (DIC) optics. Descriptions, nomenclature, and illustrations of taxonomic novelties are deposited in MycoBank [22].

### 2.3. DNA Extraction, PCR Amplification, and Sequencing

Genomic DNA was extracted from colonies grown on cellophane-covered PDA using a CTAB [cetyltrimethylammonium bromide] method [23]. For PCR amplifications of phylogenetic markers, three different primer pairs were used [19]. The PCR conditions were: an initial denaturation step of 5 min at 94 °C followed by 35 cycles of 30 s at 94 °C, 50 s at 48 °C (ITS), 54 °C (*tef-1α*), or 55 °C (*tub2*), and 1 min at 72 °C, and a final elongation step of 7 min at 72 °C. PCR amplification products were assayed via electrophoresis in 2% agarose gels. DNA sequencing was performed using an ABI PRISM^®^ 3730XL DNA Analyzer with a BigDye Terminater Kit v.3.1 (Invitrogen, Waltham, MA, USA) at the Shanghai Invitrogen Biological Technology Company Limited (Beijing, China).

### 2.4. Phylogenetic Analyses

The quality of our amplified nucleotide sequences was checked and combined by SeqMan v.7.1.0 and reference sequences (Table 1) were retrieved from the National Center for Biotechnology Information (NCBI), according to recent publications of the genus [19,20,21]. Sequences were aligned using MAFFT v. 6 [24] and manually corrected using Bioedit 7.0.9.0 [25]. Phylogenetic analyses were carried out with maximum likelihood analysis (ML), which was performed at the CIPRES web portal [26], 1000 rapid bootstrap replicates were run with GTRGAMMA model of nucleotide evolution. Bayesian inference analysis (BI) was performed in MrBayes v. 3.2.0 [27,28]. The best-fit nucleotide substitution models for each gene were selected using jModelTest v. 2.1.7 [29] under the Akaike Information Criterion. GTR + I model was selected a best-fit model for the ITS (*Neopestalotiopsis*), HKY + I + G was selected as the best-fit model for the ITS (*Pestalotiopsis*), GTR + I + G model was selected as the best-fit model for the β-tubulin, HKY + G was selected as the best-fit model for the *tef-1α*. Phylogenetic trees were viewed in FigTree v1.4. The names of the isolates from the present study are marked in blue in the trees. Maximum likelihood bootstrap support values ≥50% (BT) and Bayesian posterior probabilities ≥0.90 (PP) are given at the nodes, respectively. Alignment and trees were deposited in TreeBASE (submission ID: S29114 and S29115).

### 2.5. Pathogenicity Testing

Young and healthy leaves of *Camellia oleifera* were collected from trees growing in the greenhouse. The leaves were washed with tap water, then submerged in 70% ethanol for 2 min, and finally rinsed in sterilized water twice. The petioles of leaves were wrapped with damp cotton wool and the leaves were placed into petri dishes, three leaves per dish. One piercing wounds of each leaf were made in the mid-region forming a tiny little dot using a sterilized needle. Three drops of 6 μL spore suspension (10^6^ conidia/mL) were individually placed directly onto the leaf upper surfaces. For the control group, 6 μL of sterilized water was used. Each set of three leaves per petri dish was incubated with a different isolate. The petri dishes were placed inside a plastic box and the leaves incubated at 25 °C with humidity and 12/12 h fluorescent light/dark cycle. After 5 d, the leaves were examined for symptom development, and the diameter of diseased spot was measured.

## 3. Results

### 3.1. Phylogenetic Analyses

**Figure 1:**
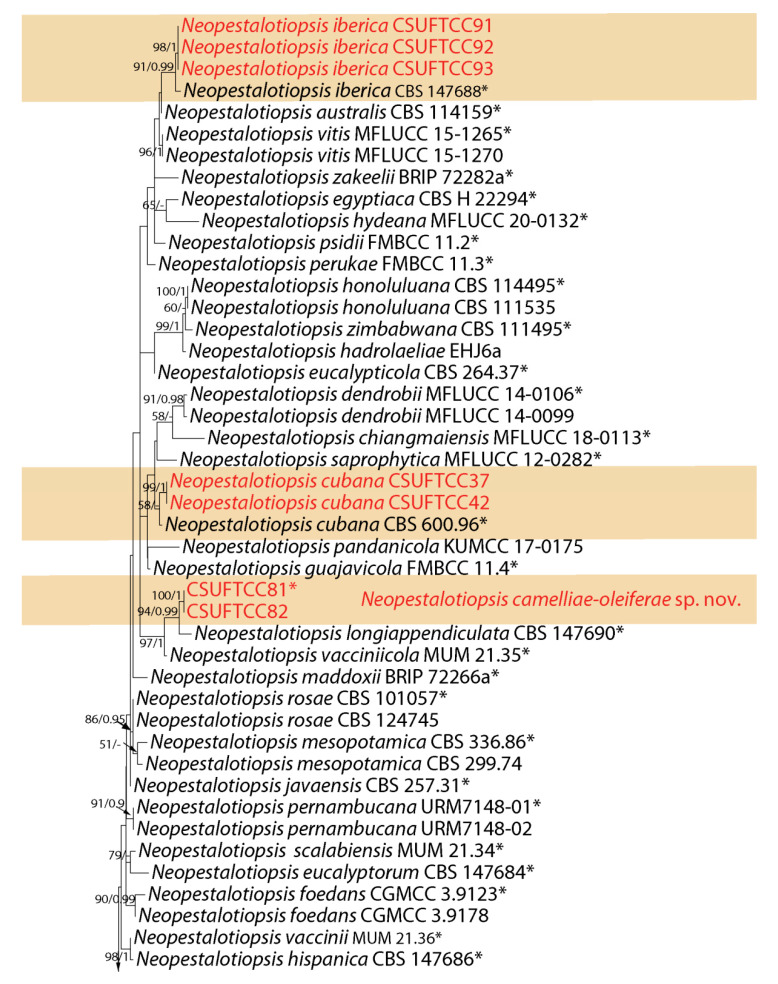

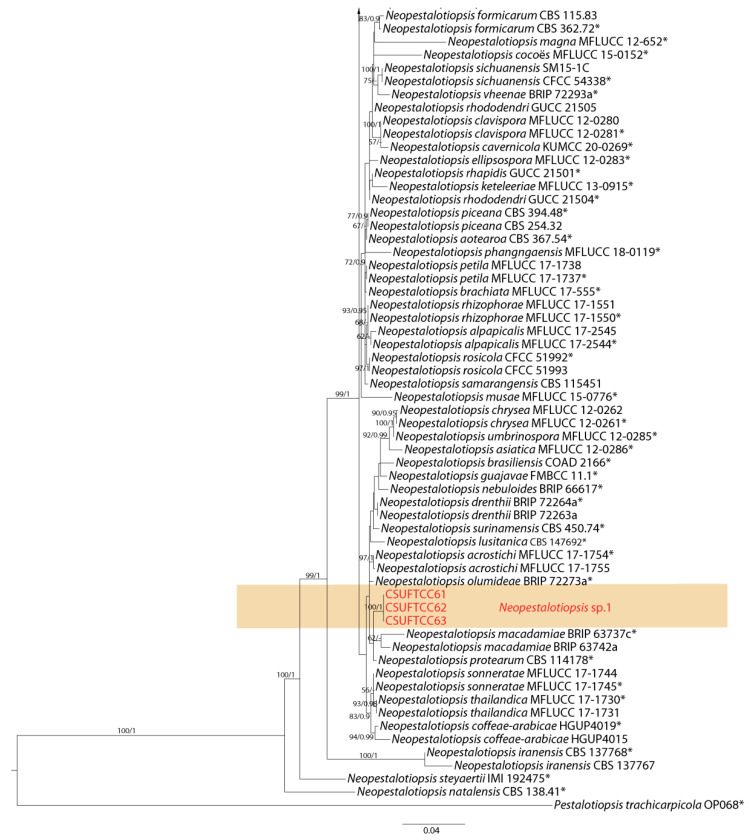
Phylogram generated from RAxML analysis based on combined ITS, *tef-1α* and *tub2* sequence data of *Neopestalotiopsis* isolates. The tree was rooted to *Pestalotiopsis trachicarpicola* (OP068). The scale bar indicates 0.04 nucleotide changes per site. Isolates from this study are marked in red and the identified species is marked in yellow. Ex-type strains are labeled with *.

The first sequence datasets for the ITS, *tef-1α* and *tub2*, were analyzed in combination to infer the interspecific relationships within Neopestalotiopsis. The combined species phylogeny of the Neopestalotiopsis isolates consisted of 105 sequences, including the outgroup Pestalotiopsis trachicarpicola (culture OP068). A total of 1389 characters including gaps (479 for ITS, 498 for *tef-1α*, and 412 for *tub2*) were included in the phylogenetic analysis. Similar tree topologies were obtained by ML and BI methods, and the best scoring ML tree is shown in Figure 1. ML bootstrap values and BI posterior probabilities (MLBS/BIPP) are given at nodes of the phylogram (Figure 1). The phylogenetic tree inferred from the concatenated alignment resolved the ten Neopestalotiopsis isolates from symptomatic leaves of *Camellia oleifera* into four well-supported monophyletic clades that represent one novel species, one undetermined species and two known species of Neopestalotiopsis (Figure 1).

**Figure 2:**
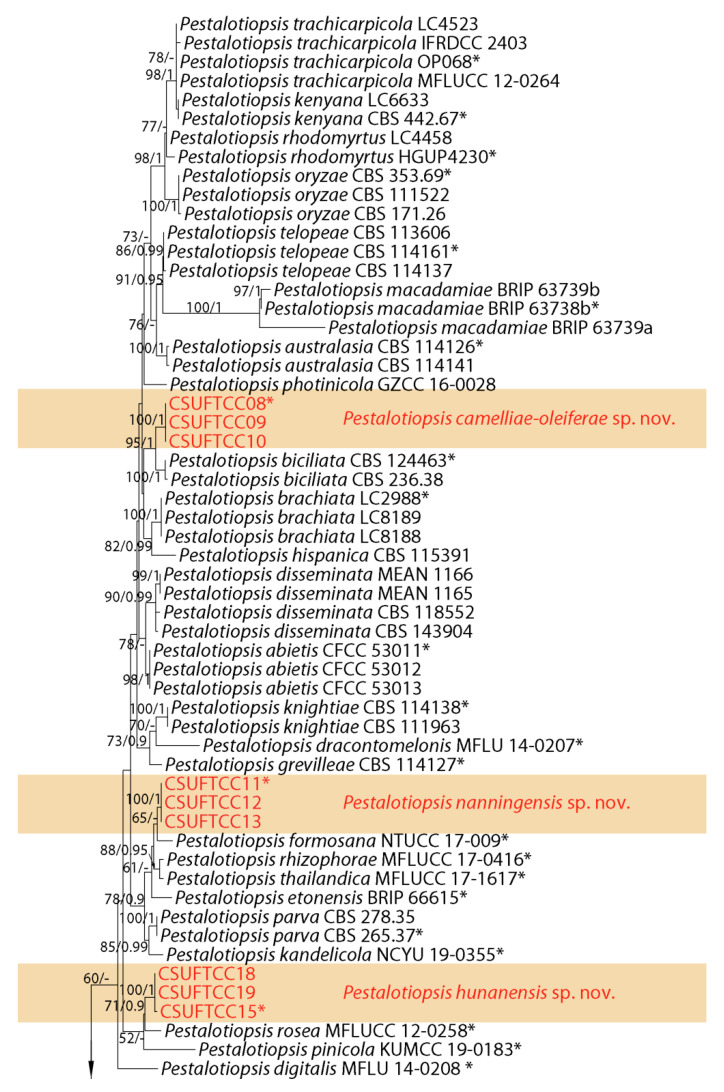

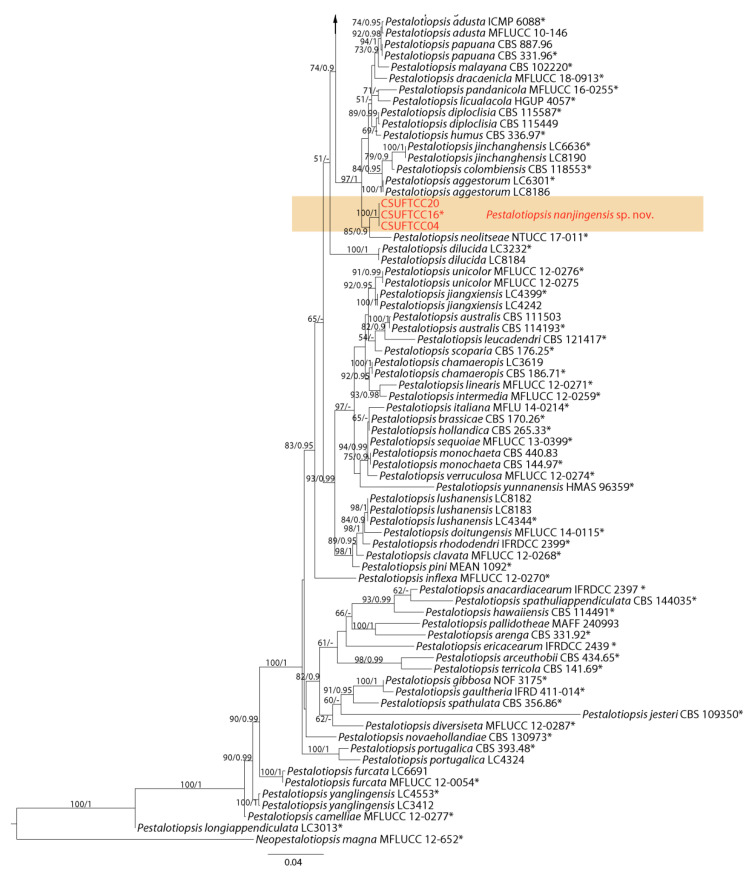
Phylogram generated from RAxML analysis based on combined ITS, *tef-1α* and *tub2* sequence data of *Pestalotiopsis* isolates. The tree was rooted to *Neopestalotiopsis magna* (MFLUCC 12-652). The scale bar indicates 0.04 nucleotide changes per site. Isolates from this study are marked in red and the identified species is marked in yellow. Ex-type strains are labeled with *.

The second sequence datasets for the ITS, *tef-1α* and *tub2* were analyzed in combination to infer the interspecific relationships within Pestalotiopsis. The combined species phylogeny of the Pestalotiopsis isolates consisted of 129 sequences, including the outgroup Neopestalotiopsis magna (culture MFLUCC 12-652). A total of 1557 characters including gaps (515 for ITS, 537 for *tef-1α*, and 505 for *tub2*) were included in the phylogenetic analysis. Similar tree topologies were obtained by ML and BI methods, and the best scoring ML tree is shown in Figure 2. ML bootstrap values and BI posterior probabilities (MLBS/BIPP) are given at nodes of the phylogram (Figure 2). The phylogenetic tree inferred from the concatenated alignment resolved the 12 Pestalotiopsis isolates from symptomatic leaves of *Camellia oleifera* into four well-supported monophyletic clades that represent four novel species of Pestalotiopsis (Figure 2).

### 3.2. Taxonomy

**Figure 3:**
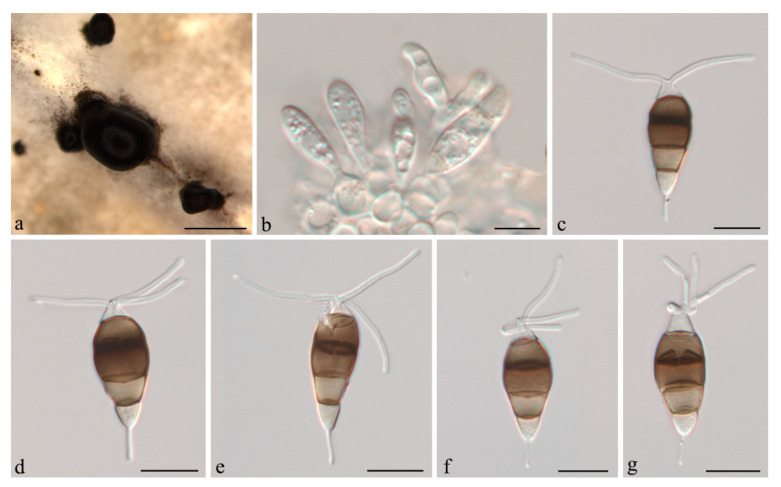
*Neopestalotiopsis camelliae-oleiferae* (CSUFTCC81). (**a**) Conidioma formed on PDA, (**b**) conidiogenous cells, and (**c**–**g**) conidia. Scale bars: (**a**) = 1 mm, (**b**–**g**) = 10 μm.

***Neopestalotiopsis camelliae-oleiferae*** Q. Yang & H. Li, **sp. nov.** (Figure 3).

**MycoBank**: MB841476.

**Etymology:** Named after the host species, *Camellia oleifera*.

**Holotype:** CSUFT081.

**Description:***Conidiomata acervular* in culture on PDA, globose, 300–800 μm diam., solitary or aggregated in clusters, exuding black conidial masses. Conidiophores reduced to conidiogenous cells. *Conidiogenous cells *ampulliform, hyaline, smooth, annelidic. *Conidia* fusiform to clavate, straight or slightly curved, 22.5–24(−26.5) × (7–)8.5–10 μm, 4-septate; basal cell conical, 3.5–4.5 μm, hyaline or sometimes pale brown, smooth, thin-walled; with a single appendage filiform, unbranched, centric, (4.5–)6–8(−9) μm long; three median cells doliiform, 14–16(−18) μm long, smooth, versicoloured, septa darker than the rest of the cell (second cell from base pale brown, 4.5–5.5 μm long; third cell medium to dark brown, 5–5.5(−6.5) μm long; fourth cell medium to dark brown, 4.5–6 μm long); apical cell conical, 2.5–4.5 μm long, hyaline, smooth, thin-walled; with 2–3 apical tubular appendages unbranched, filiform, (13.5–)15.5–18.5(−20.5) μm long. *Sexual morph *not observed.

**Culture characteristics:** Colonies on PDA reaching 55 mm diameter after seven days at 25 °C. Colonies filamentous to circular, with dense aerial mycelium on surface, fruiting bodies black.

**Material examined:** CHINA, Jiangsu Province, Nanjing City, from leaf spots of *Camellia oleifera*, 25 Oct. 2020, H. Li (CSUFT081, holotype); ex-type living culture CSUFTCC81, living culture CSUFTCC82.

**Notes:***Neopestalotiopsis camelliae-oleiferae* was collected from symptomatic leaves of *C. oleifera* in Jiangsu Province, China. Two isolates (CSUFTCC81 and CSUFTCC82) representing *N. camelliae-oleiferae* clustered in a well-support clade (ML/BI = 100/1). *Neopestalotiopsis camelliae-oleiferae* was sister to a clade containing *N. longiappendiculata *and N. vacciniicola. *N. camelliae-oleiferae* can be distinguished from *N. longiappendiculata *based on ITS, *tef-1α * and *tub2* loci (3/449 in ITS, 3/450 in *tef-1α *, and 6/404 in *tub2*, no gaps). Morphologically, *N. camelliae-oleiferae* differs from *N. longiappendiculata *by wider conidia (8.5–10 vs. 7–7.8 μm); from N. vacciniicola by shorter apical tubular appendages (15.5–18.5 vs. 25.7–30.2 μm) [20]. Therefore, the collection in the present study is designated as a new species.

**Figure 4:**
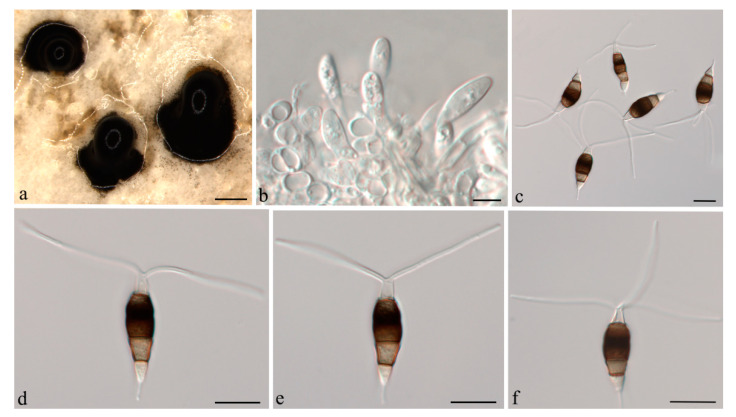
*Neopestalotiopsis cubana* (CSUFTCC37). (**a**) Conidiomata formed on PDA, (**b**) conidiogenous cells, and (**c**–**f**) conidia. Scale bars: (**a**) = 500 μm, (**b**–**f**) = 10 μm.

***Neopestalotiopsis cubana*** Maharachch, K.D. Hyde & Crous, in Maharachchikumbura, Hyde, Groenewald, Xu & Crous, Stud. Mycol. 79: 138 (2014) (Figure 4).

**Description:***Conidiomata acervular* in culture on PDA, globose, 800–1350 μm diam., solitary or aggregated in clusters, exuding black conidial masses. Conidiophores reduced to conidiogenous cells. *Conidiogenous cells *ampulliform to cylindrical, hyaline, smooth, annelidic. *Conidia* fusoid to ellipsoidal, straight or slightly curved, (19.5–)21–25(−26.5) × (5.5–)6.5–8 μm, 4-septate; basal cell conical, 3.5–4.5 μm, hyaline or sometimes pale brown, smooth, thin-walled; with a single appendage filiform, unbranched, centric, 3–5.5 μm long; three median cells doliiform, 13.5–15(−16) μm long, smooth, versicoloured, septa darker than the rest of the cell (second cell from base pale brown, 3.5–5.5 μm long; third cell medium to dark brown, 4–5 μm long; fourth cell medium to dark brown, 3.5–4.5 μm long); apical cell conical, 3.5–4.5 μm long, hyaline, smooth, thin-walled; with 2–3 apical tubular appendages, unbranched, filiform, (21–)24–29(−31) μm long. *Sexual morph *not observed.

**Culture characteristics:** Colonies on PDA reaching 70 mm diameter after seven days at 25 °C. Colonies filamentous to circular, medium dense, aerial mycelium on surface flat or raised, pycnidia abundant, fruiting bodies black.

**Material examined:** CHINA, Hainan Province, Chengmai County, from leaf spots of *Camellia oleifera*, 9 Nov. 2020, H. Li (CSUFT042); living cultures CSUFTCC37 and CSUFTCC42.

**Notes:***Neopestalotiopsis cubana* was originally described from leaf litter in Cuba [8]. In the present study, two isolates from leaves of symptomatic *C. oleifera* were congruent with *N. cubana* based on morphology and DNA sequences data (Figure 1). We therefore describe *N. cubana* as a known species for this clade.

**Figure 5:**
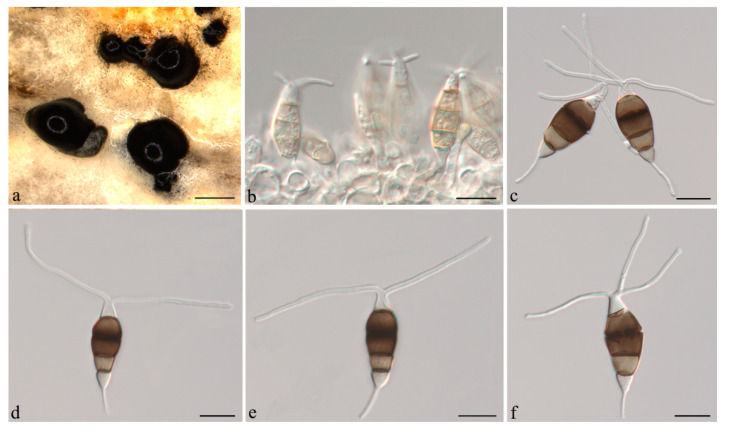
*Neopestalotiopsis iberica* (CSUFTCC91). (**a**) Conidiomata formed on PDA, (**b**) conidiogenous cells, and (**c**–**f**) conidia. Scale bars: (**a**) = 1 mm, (**b**–**f**) = 10 μm.

***Neopestalotiopsis iberica*** E. Diogo, M.H. Bragança & A.J.L. Phillips, in Diogo, Gonçalves, Silva, Valente, Bragança & Phillips, *Mycol. Progr. *20(11): 1449 (2021) (Figure 5).

**Description:***Conidiomata acervular* in culture on PDA, globose, 600–1500 μm diameter, solitary or aggregated in clusters, exuding black conidial masses. Conidiophores reduced to conidiogenous cells. *Conidiogenous cells *ampulliform, hyaline, smooth, annelidic. *Conidia* fusiform to ellipsoidal, straight or slightly curved, (21.5–)22.5–24(−26.5) × 7–9(−10.5) μm, 4-septate; basal cell conical, 3.5–4.5 μm, hyaline or sometimes pale brown, smooth, thin-walled; with a single appendage filiform, unbranched, centric, 2.5–4 μm long; three median cells doliiform, 12.5–14.5(−15.5) μm long, smooth, versicoloured, septa darker than the rest of the cell (second cell from base pale brown, 4.5–5 μm long; third cell medium to dark brown, 4.5–5.5(−6) μm long; fourth cell medium to dark brown, 4.5–5.5 μm long); apical cell conical, 2.5–4 μm long, hyaline, smooth, thin-walled; with 2–3 apical tubular appendages, unbranched, filiform, 24–26(−29.5) μm long. *Sexual morph *not observed.

**Culture characteristics:** Colonies on PDA reaching 70 mm diameter after seven days at 25 °C. Colonies filamentous to circular, medium dense, aerial mycelium on surface flat or raised, with filiform margin, fluffy, fruiting bodies black.

**Material examined:** CHINA, Jiangsu Province, Nanjing City, from leaf spots of *Camellia oleifera*, 25 Oct. 2020, H. Li (CSUFT091); living cultures LHNJ91, LHNJ92, and LHNJ93.

**Notes:***Neopestalotiopsis iberica* was originally described from leaves and stems of *Eucalyptus globulus* in Portugal [30]. In the present study, three isolates from leaves of symptomatic *C. oleifera* were congruent with *N. iberica* based on morphology and DNA sequences data (Figure 1). We therefore describe *N. iberica* as a known species for this clade.

**Figure 6:**
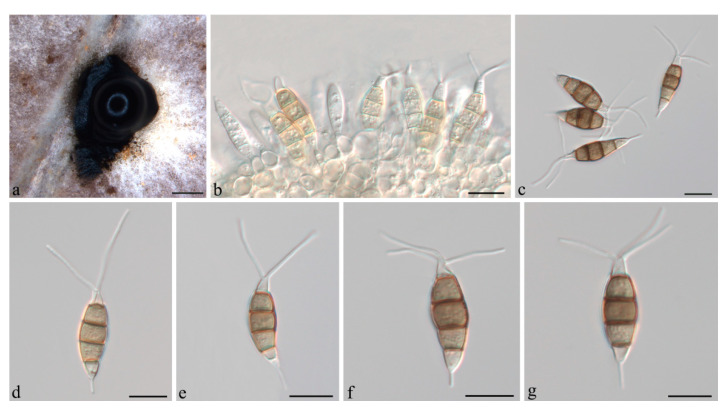
*Pestalotiopsis camelliae-oleiferae *(CSUFTCC08). (**a**) Conidioma formed on PDA, (**b**) conidiogenous cells, and (**c**–**g**) conidia. Scale bars: (**a**) = 1 mm, (**b**–**g**) = 10 μm.

***Pestalotiopsis camelliae-oleiferae *** Q. Yang & H. Li, **sp. nov.** (Figure 6).

MycoBank: MB841478.

**Etymology:** Named after the host species, *Camellia oleifera*.

**Holotype:** CSUFT008.

**Description:***Conidiomata acervular* in culture on PDA, globose, 1.0–2.6 mm diameter, solitary or aggregated in clusters, exuding black conidial masses. Conidiophores reduced to conidiogenous cells. *Conidiogenous cells *discrete or integrated, cylindrical to subcylindrical, hyaline, smooth. *Conidia* fusoid, ellipsoid, straight or slightly curved, (19.5–)21.5–23(−25) × (5–)6–7 μm, 4-septate; basal cell conic to obconic with a truncate base, 3.5–5.5 μm, hyaline, smooth, thin-walled; with a single appendage filiform, unbranched, centric, 2.5–4.5 μm long; three median cells doliiform, 12.5–14 μm long, smooth, concolorous, brown, septa darker than the rest of the cell (second cell from base 4–4.5 μm long; third cell 4.5–5 μm long; fourth cell 3.5–4.5 μm long); apical cell conical, 2.5–4(−4.5) μm long, hyaline, smooth, thin-walled; with 2–3 apical tubular appendages, unbranched, filiform, (11–)12.5–14.5(−16) μm long. *Sexual morph *not observed.

**Culture characteristics:** Colonies on PDA reaching 70 mm diameter after seven days at 25 °C. Colonies filamentous to circular, medium dense, with white sparse mycelium, fruiting bodies black.

**Material examined:** CHINA, Hunan Province, Changsha City, from leaf spots of *Camellia oleifera*, 30 Aug. 2020, H. Li (CSUFT008, holotype); ex-type living culture CSUFTCC08, living cultures CSUFTCC09 and CSUFTCC10.

**Notes:***Pestalotiopsis camelliae-oleiferae *was sister to *P. biciliata * in a well-supported clade (ML/BI = 100/1) (Figure 2). *Pestalotiopsis camelliae-oleiferae *can be distinguished from *P. biciliata * based on ITS, *tef-1α* and *tub2* loci (4/500 in ITS, 1/473 in*tef-1α *, and 6/443 in *tub2*, no gaps). Morphologically, *P. camelliae-oleiferae *differs from *P. biciliata * by shorter conidia (21.5–23 vs. 22–28 μm) [8]. Therefore, the collection in the present study is designated as a new species.

**Figure 7:**
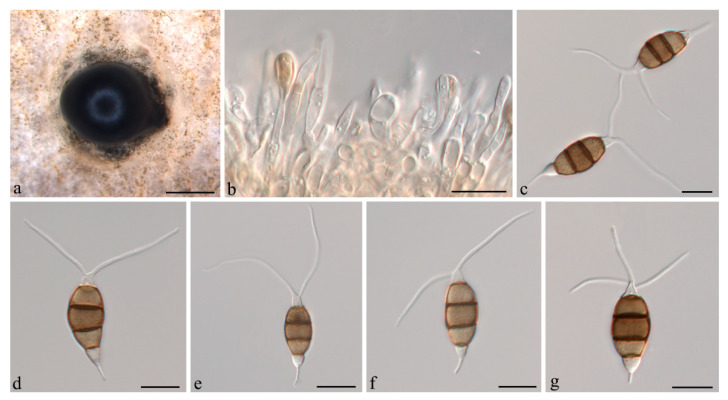
*Pestalotiopsis hunanensis * (CSUFTCC15). (**a**) Conidioma formed on PDA, (**b**) conidiogenous cells, and (**c**–**g**) conidia. Scale bars: (**a**) = 1 mm, (**b**–**g**) = 10 μm.

***Pestalotiopsis hunanensis *** Q. Yang & H. Li, **sp. nov.** (Figure 7).

MycoBank: MB841480.

**Etymology:** In reference to the Hunan Province, from where the fungus was first collected.

**Holotype:** CSUFT015.

**Description:***Conidiomata acervular* in culture on PDA, globose, 500–1000 μm diameter, solitary or aggregated in clusters, exuding black conidial masses. Conidiophores reduced to conidiogenous cells. *Conidiogenous cells *discrete or integrated, cylindrical to subcylindrical, hyaline, smooth, annelidic. *Conidia* fusoid, ellipsoid, straight or slightly curved, (20.5–)23–25(−26.5) × (7–)9–10.5 μm, 4-septate; basal cell conic to obconic with a truncate base, 4–5.5 μm, hyaline, smooth, thin-walled; with a single appendage filiform, unbranched, centric, 3–3.5 μm long; three median cells doliiform, (14–)15–18 μm long, smooth, concolorous, brown, septa darker than the rest of the cell (second cell from base 4–5 μm long; third cell 5–6.5 μm long; fourth cell 4.5–5.5 μm long); apical cell conical, 2.5–3 μm long, hyaline, smooth, thin-walled; with 2–3 apical tubular appendages, unbranched, filiform, (13.5–)15–22(−26.5) μm long. *Sexual morph *not observed.

**Culture characteristics:** Colonies on PDA reaching 50 mm diameter after seven days at 25 °C. Colonies filamentous to circular, with sparse aerial mycelium, fruiting bodies black.

**Material examined:** CHINA, Hunan Province, Xiangtan City, from leaf spots of *Camellia oleifera*, 7 Nov. 2020, H. Li (CSUFT015, holotype); ex-type living culture CSUFTCC15, living cultures CSUFTCC18 and CSUFTCC19.

**Notes:***Pestalotiopsis hunanensis * was sister to *P. rosae * in a well-supported clade (ML/BI = 100/1) (Figure 2). *Pestalotiopsis hunanensis * can be distinguished from P. rosea based on ITS, *tef-1α* and *tub2* loci (6/501 in ITS, 13/475 in *tef-1α*, and 7/446 in *tub2*, 12 gaps). Morphologically, *P. hunanensis * differs from *P. rosae * by lager conidia (23–25 × 9–10.5 vs. 17.5–21.8 × 5.7–7 μm) [6]. Therefore, the collection in the present study is designated as a new species.

**Figure 8:**
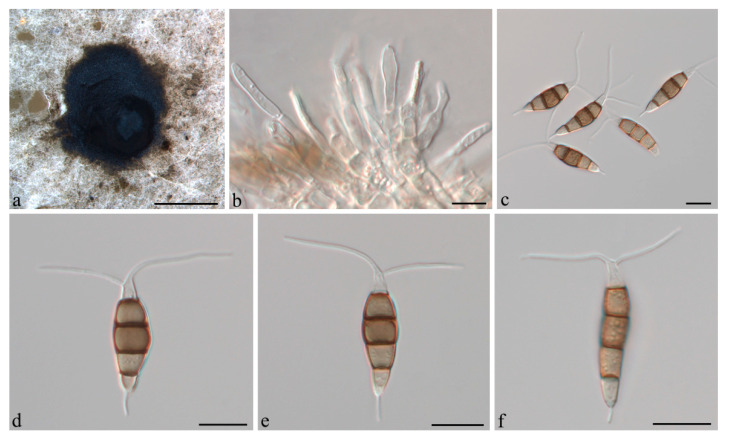
*Pestalotiopsis nanjingensis *(CSUFTCC16). (**a**) Conidioma formed on PDA, (**b**) conidiogenous cells, and (**c**–**f**) conidia. Scale bars: (**a**) = 1 mm, (**b**–**f**) = 10 μm.

***Pestalotiopsis nanjingensis*** Q. Yang & H. Li, **sp. nov.** (Figure 8).

MycoBank: MB841481.

**Etymology:** In reference to the Nanjing City, from where the fungus was first collected.

**Holotype:** CSUFT016.

**Description:***Conidiomata acervular* in culture on PDA, globose, 1000–1600 μm diameter, solitary or aggregated in clusters, exuding black conidial masses. Conidiophores reduced to conidiogenous cells. *Conidiogenous cells *discrete or integrated, cylindrical to subcylindrical, hyaline, smooth, annelidic.* Conidia* fusoid, ellipsoid, straight or slightly curved, (19.5–)22–25 × (4.5–)5–6.5 μm, 4-septate; basal cell conic to obconic with a truncate base, 4.5–5 μm, hyaline, smooth, thin-walled; with a single appendage filiform, unbranched, centric, 2.5–3.5 μm long; three median cells doliiform, 13–14.5(−16) μm long, smooth, concolorous, brown, septa darker than the rest of the cell (second cell from base 4.5–5.5 μm long; third cell 4.5–5.5 μm long; fourth cell 3.5–4.5 μm long); apical cell conical, 3.5–4 μm long, hyaline, smooth, thin-walled; with two apical tubular appendages, unbranched, filiform, (11–)13.5–18(−20) μm long. *Sexual morph *not observed.

**Culture characteristics:** Colonies on PDA reaching 60 mm diameter after seven days at 25 °C. Colonies filamentous to circular, medium dense, aerial mycelium on surface flat, fruiting bodies black.

**Material examined:** CHINA, Jiangsu Province, Nanjing city, from leaf spots of *Camellia oleifera*, 25 Oct. 2020, H. Li (CSUFT016, holotype); ex-type living culture CSUFTCC 16, living cultures CSUFTCC04 and CSUFTCC20.

**Notes:***Pestalotiopsis nanjingensis *was sister to P. neolitseae in a well-supported clade (ML/BI = 100/1) (Figure 2). *Pestalotiopsis nanjingensis *can be distinguished from P. neolitseae based on ITS, *tef-1α* and *tub2* loci (2/500 in ITS, 26/472 in *tef-1α*, and 2/442 in *tub2*, 5 gaps). Morphologically, P. nanjingensis differs from P. neolitseae by longer conidia (22–25 vs. 18–21 μm) and apical appendages (13.5–18 vs. 10–15 μm) [15]. Therefore, the collection in the present study is designated as a new species.

**Figure 9:**
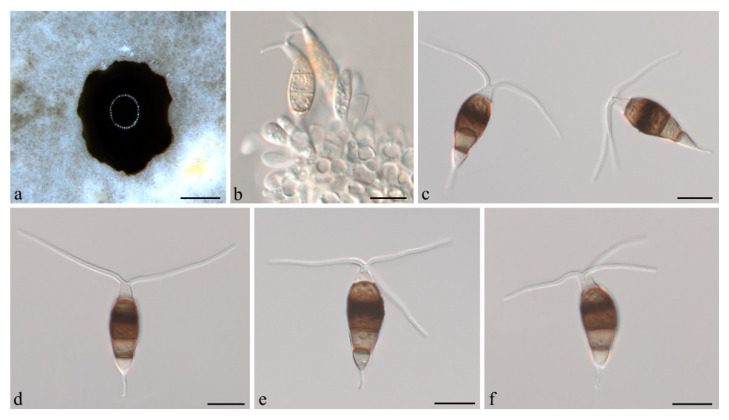
Pestalotiopsis nanningensis (CSUFTCC10). (**a**) Conidioma formed on PDA, (**b**) conidiogenous cells, and (**c**–**f**) conidia. Scale bars: (**a**) = 500 μm, (**b**–**f**) = 10 μm.

***Pestalotiopsis nanningensis*** Q. Yang & H. Li, **sp. nov.** (Figure 9).

MycoBank: MB841479.

**Etymology:** In reference to the Nanning City, from where the fungus was first collected.

**Holotype:** CSUFT011.

**Description:***Conidiomata acervular* in culture on PDA, globose, 750–1200 μm diameter, solitary or aggregated in clusters, exuding black conidial masses. *Conidiophores* reduced to conidiogenous cells. *Conidiogenous cells *discrete or integrated, cylindrical to subcylindrical, hyaline, smooth, annelidic. *Conidia* fusoid, ellipsoid, straight or slightly curved, (22–)24–26.5 × (6–)7–8(−9) μm, 4-septate; basal cell conical, 4.5–6 μm, hyaline, smooth, thin-walled; with a single appendage filiform, unbranched, centric, 4.5–6.5 μm long; three median cells doliiform, 13.5–15(−17) μm long, smooth, concolorous, brown, septa darker than the rest of the cell (second cell from base 4.5–5.5 μm long; third cell 5–6 μm long; fourth cell 4–5 μm long); apical cell conical, 3.5–4.5 μm long, hyaline, smooth, thin-walled; with 2–3 apical tubular appendages, unbranched, filiform, (13.5–)18–22.5(−26.5) μm long. *Sexual morph* not observed.

**Culture characteristics:** Colonies on PDA reaching 80 mm diameter after seven days at 25 °C. Colonies filamentous to circular, medium dense, white aerial mycelium on surface flat or raised.

**Material examined:** CHINA, Guangxi Province, Nanning City, from leaf spots of *Camellia oleifera*, 20 Oct. 2020, H. Li (CSUFT011, holotype); ex-type living culture CSUFTCC11, living cultures CSUFTCC12 and CSUFTCC13.

**Notes:*** Pestalotiopsis nanningensis* was sister to P. formosana in a well-supported clade (ML/BI = 100/1) (Figure 2).* Pestalotiopsis nanningensis* can be distinguished from P. formosana based on ITS and *tef-1α * loci (4/500 in ITS, 2/472 in *tef-1α *, and 1/442 in *tub2*, no gaps). Morphologically, P. nanningensis differs from P. formosana by lager conidia (24–26.5 × 7–8 vs. 18–22 × 6–7 μm) and longer apical appendages (18–22.5 vs. 11–16 μm) [15]. Therefore, the collection in the present study is designated as a new species.

### 3.3. Pathogenicity Assay

**Figure 10:**
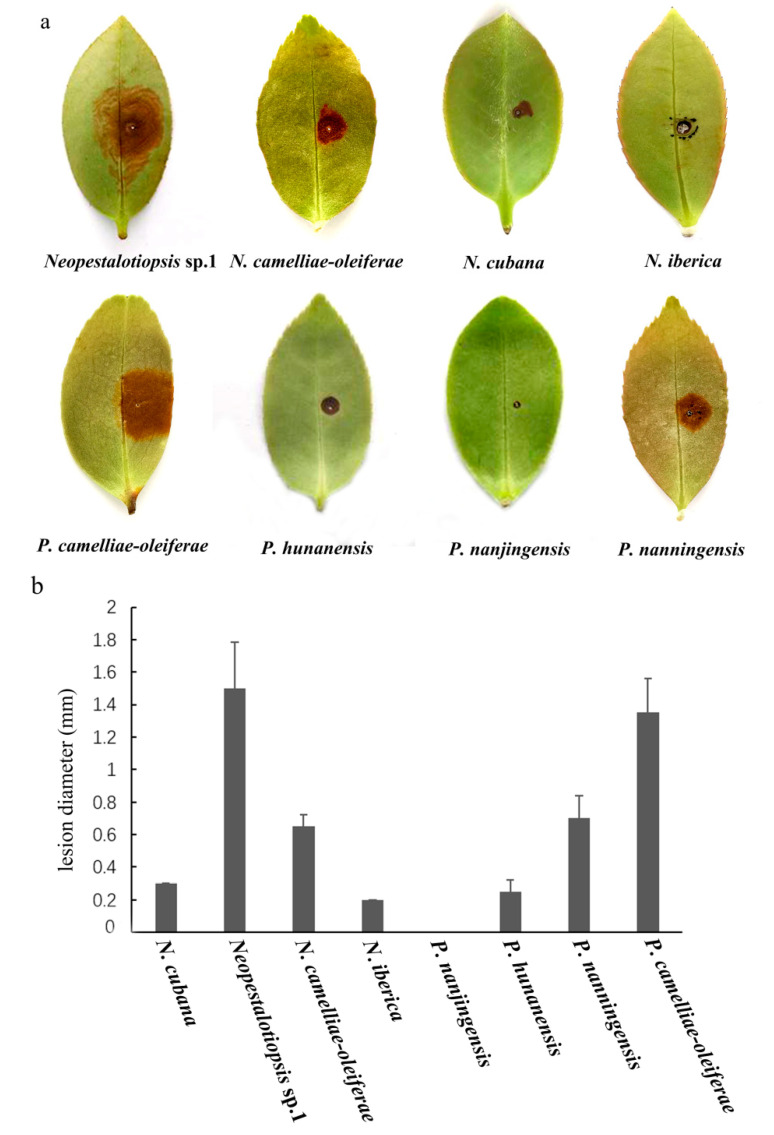
Pathogenicity of eight pestalotioid species from tea-oil leaves. (**a**) Induced symptoms on tea-oil leaves after 5 days. (**b**). The virulence of the isolates was evaluated by measuring the diameters of the necrotic lesions on infected tea-oil leaves 5 days after wounding.

After five days, for the pathogenicity tests, *N. camelliae-oleiferae*, *N. cubana*, *N. iberica* *Neopestalotiopsis* sp.1, *P. camelliae-oleiferae*, *P. hunanensis *, and *P. nanningensis* developed brown lesions on wounded leaves (right), whereas the controls showed no symptoms (left). *Neopestalotiopsis* sp.1 had the highest virulence, while *P. nanjingensis* did not cause obvious symptoms (Figure 10). Koch’s postulates were fulfilled by reisolating the same fungi and verifying its colony and morphological characters.

## 4. Discussion

In this study, an investigation of *C. oleifera* diseases in China was carried out and Camellia leaf disease caused by pestalotioid fungi was observed as a common disease. Identification of our collections was conducted, based on isolates from symptomatic leaves of *C. oleifera* using three combined loci (ITS, *tef-1α* and *tub2*), as well as morphological characteristics. It includes *N. cubana*, *N. iberica,* as well as five new species named *N. camelliae-oleiferae*, *P. camelliae-oleiferae*, *P. hunanensis*, *P. nanjingensis,* and *P.* *nanningensis*.

The expanding cultivation of *C. oleifera* over the last several decades has attracted increasing attention from plant pathologists to infectious diseases on this crop. Therein, pestalotioid species are more frequently regarded as endophytes or latent pathogens causing diseases only on specific situations [4,6,12,63,64]. Understanding the diversity of pestalotioid species and the genetic variation within pathogen populations could help in developing sustainable disease management strategies.

Pestalotioid fungi (Pestalotiopsidaceae, Sordariomycetes) are species-rich asexual taxa, which are common pathogens that cause a variety of diseases, including leaf spots, shoot dieback, fruit rots and various post-harvest diseases [6,8,15,19,20,46,65]. As many peatalotioid species have overlapping morphological traits, sequence data is essential to resolve these three genera and introduce new species [8]. Combined gene sequence of ITS, *tef-1α*, and *tub2* can provide a better resolution for *Pestalotiopsis* and *Pseudopestalotiopsis*. However, more genes are needed to provide better resolution and support in *Neopestalotiopsis*. Furthermore, this is the first systematic report of *Neopestalotiopsis* and *Pestalotiopsis* fungi associated with *Camellia oleifera* in China, which indicates that there may be a high undescribed diversity of fungi in this host.

Pathogenicity tests of eight pestalotioid species from *Camellia oleifera* showed that all species except for *P. nanjingensis* were capable of infecting wounded leaves. *Neopestalotiopsis* sp.1 and *P. camelliae-oleiferae* showed stronger virulence, with lesion diameters ranged from 14.7 to 17.8 mm on leaves of the *Neopestalotiopsis* sp.1 isolate (CSUFTCC61) and 13.5 to 15.5 mm on leaves of the *P. camelliae-oleiferae* isolate (CSUFTCC08). All pathogenicity tests were performed with a single *C. camellia* cultivar. Since different *C. oleifera* cultivars may have different resistance to pestalotioid species, more cultivars of *C. oleifera* should be studied for the variation of their resistance to pestalotioid pathogens. During the tests, the symptoms vary considerably with factors, such as relative humidity, temperature, and the inoculum concentration. In the future, field conditions with natural inoculum should be conducted rather than just in vitro artificial inoculation.

## 5. Conclusions

Seven peatalotioid species (two known species and five new species) were described and illustrated. This is the first systematic report of *Neopestalotiopsis* and *Pestalotiopsis* fungi associated with *Camellia oleifera* in China. The pathogenicity of these species on leaves were examined and showed that there were significant differences in the pathogenicity.

## Figures and Tables

**Table 1:** Isolates and GenBank accession numbers of sequences used in this study.

Species	Isolate	Host/Substrate	Location	GenBank Accessions Numbers			
				ITS	*tub2*	*tef-1α*	References
*Neopestalotiopsis acrostichi*	MFLUCC 17-1754 *	*Acrostichum aureum*	Thailand	MK764272	MK764338	MK764316	[19]
	MFLUCC 17-1755	*Acrostichum aureum*	Thailand	MK764273	MK764339	MK764317	[19]
*N. alpapicalis*	MFLUCC 17-2544 *	*Rhizophora mucronata*	Thailand	MK357772	MK463545	MK463547	[30]
	MFLUCC 17-2545	*Symptomatic Rhizophora*	Thailand	MK357773	MK463546	MK463548	[30]
*N. aotearoa*	CBS 367.54 *	Canvas	New Zealand	KM199369	KM199454	KM199526	[6]
*N. asiatica*	MFLUCC 12-0286 *	*Prunus dulcis*	China	JX398983	JX399018	JX399049	[8]
*N. australis*	CBS 114159 *	*Telopea* sp.	Australia	KM199348	KM199432	KM199537	[8]
*N. brachiata*	MFLUCC 17-1555 *	*Rhizophora apiculata*	Thailand	MK764274	MK764340	MK764318	[19]
*N. brasiliensis*	COAD 2166 *	*Psidium guajava*	Brazil	MG686469	MG692400	MG692402	[31]
*N. camelliae-oleiferae*	CSUFTCC81 *	*Camellia oleifera*	China	OK493585	OK562360	OK507955	This study
	CSUFTCC82	*Camellia oleifera*	China	OK493586	OK562361	OK507956	This study
*N. cavernicola*	KUMCC 20-0269 *	Cave	China	MW545802	MW557596	MW550735	[32]
*N. chiangmaiensis*	MFLUCC 18-0113 *	*Pandanus* sp.	Thailand	NA	MH412725	MH388404	[18]
*N. chrysea*	MFLUCC 12-0261 *	Dead leaves	China	JX398985	JX399020	JX399051	[6]
	MFLUCC 12-0262	Dead leaves	China	JX398986	JX399021	JX399052	[6]
*N. clavispora*	MFLUCC 12-0281 *	*Magnolia* sp.	China	JX398979	JX399014	JX399045	[6]
	MFLUCC 12-0280	*Magnolia* sp.	China	JX398978	JX399013	JX399044	[6]
*N. cocoës*	MFLUCC 15-0152 *	*Cocos nucifera*	Thailand	NR 156312	NA	KX789689	[19]
*N. coffeae-arabicae*	HGUP4015	*Coffea arabica*	China	KF412647	KF412641	KF412644	[33]
	HGUP4019 *	*Coffea arabica*	China	KF412649	KF412643	KF412646	[33]
*N. cubana*	CBS 600.96 *	Leaf litter	Cuba	KM199347	KM199438	KM199521	[8]
	CSUFTCC37	*Camellia oleifera*	China	OK493583	OK562358	OK507953	This study
	CSUFTCC42	*Camellia oleifera*	China	OK493584	OK562359	OK507954	This study
*N. dendrobii*	MFLUCC 14-0106 *	*Dendrobium cariniferum*	Thailand	MK993571	MK975835	MK975829	[34]
	MFLUCC 14-0099	*Dendrobium cariniferum*	Thailand	MK993570	MK975834	MK975828	[34]
*N. drenthii*	BRIP 72263a	*Macadamia integrifolia*	Australia	MZ303786	MZ312679	MZ344171	[21]
	BRIP 72264a *	*Macadamia integrifolia*	Australia	MZ303787	MZ312680	MZ344172	[21]
*N. egyptiaca*	CBS 1401628	*Mangifera indica*	Egypt	KP943747	KP943746	KP943748	[35]
*N. ellipsospora*	MFLUCC 12-02838	Dead plant material	China	JX398980	JX399016	JX399047	[6]
*N. eucalyptorum*	CBS 147684 *	*Eucalyptus globulus*	Portugal	MW794108	MW802841	MW805397	[20]
*N. eucalypticola*	CBS 264.37 *	*Eucalyptus globulus*	NA	KM199376	KM199431	KM199551	[8]
*N. foedans*	CGMCC 3.9123 *	Mangrove plant	China	JX398987	JX399022	JX399053	[6]
	CGMCC 3.9178	*Neodypsis decaryi*	China	JX398989	JX399024	JX399055	[6]
*N. formicarum*	CBS 362.72 *	Dead ant	Cuba	KM199358	KM199455	KM199517	[8]
	CBS 115.83	Plant debris	Cuba	KM199344	KM199444	KM199519	[8]
*N. guajavae*	FMBCC 11.1 *	Guava	Pakistan	MF783085	MH460871	MH460868	[36]
*N. guajavicola*	FMBCC 11.4 *	Guava	Pakistan	MH209245	MH460873	MH460870	[36]
*N. hadrolaeliae*	EHJ6a	*Cattleya jongheana*	Brazil	MK454709	MK465120	MK465122	[37]
*N. hispanica*	CBS 147686 *	*Eucalyptus globulus*	Portugal	MW794107	MW802840	MW805399	[20]
*N. honoluluana*	CBS 114495 *	*Telopea* sp.	USA	KM199364	KM199457	KM199548	[8]
	CBS 111535	*Telopea* sp.	USA	KM199363	KM199461	KM199546	[8]
*N. hydeana*	MFLUCC 20-0132 *	*Artocarpus heterophyllus*	Thailand	MW266069	MW251119	MW251129	[38]
*N. iberica*	CSUFTCC91	*Camellia oleifera*	China	OK493587	OK562362	OK507957	This study
	CSUFTCC92	*Camellia oleifera*	China	OK493588	OK562363	OK507958	This study
	CSUFTCC93	*Camellia oleifera*	China	OK493589	OK562364	OK507959	This study
	CBS 147688 *	*Eucalyptus globulus*	Portugal	MW794111	MW802844	MW805402	[20]
*N. iraniensis*	CBS 137768 *	*Fragaria ananassa*	Iran	KM074048	KM074057	KM074051	[39]
	CBS 137767	*Fragaria ananassa*	Iran	KM074045	KM074056	KM074053	[39]
*N. javaensis*	CBS 257.31 *	*Cocos nucifera*	Indonesia	KM199357	KM199457	KM199548	[8]
*N. keteleerie*	MFLUCC 13-0915 *	*Keteleeria pubescens*	China	KJ503820	KJ503821	KJ503822	[40]
*N. longiappendiculata*	CBS 147690 *	*Eucalyptus globulus*	Portugal	MW794110	MW802845	MW805404	[20]
*N. lusitanica*	CBS 147692 *	*Eucalyptus globulus*	Portugal	MW794112	MW802843	MW805406	[20]
*N. macadamiae*	BRIP 63737c *	*Macadamia integrifolia*	Australia	KX186604	KX186654	KX186629	[14]
	BRIP 63742a	*Macadamia integrifolia*	Australia	KX186599	KX186657	KX186627	[14]
*N. maddoxii*	BRIP 72266a *	*Macadamia integrifolia*	Australia	MZ303782	MZ312675	MZ344167	[14]
*N. magna*	MFLUCC 12-0652 *	*Pteridium* sp.	France	KF582795	KF582793	KF582791	[41]
*N. mesopotamica*	CBS 336.86 *	*Pinus brutia*	Iraq	KM199362	KM199441	KM199555	[8]
	CBS 299.74	*Eucalyptus* sp.	Turkey	KM199361	KM199435	KM199541	[8]
*N. musae*	MFLUCC 15-0776 *	*Musa* sp.	Thailand	KX789683	KX789686	KX789685	[19]
*N. natalensis*	CBS 138.41 *	*Acacia mollissima*	South Africa	KM199377	KM199466	KM199552	[8]
*N. nebuloides*	BRIP 66617 *	*Sporobolus elongatus*	Australia	MK966338	MK977632	MK977633	[42]
*N. olumideae*	BRIP 72273a *	*Macadamia integrifolia*	Australia	MZ303790	MZ312683	MZ344175	[21]
*N. pandanicola*	KUMCC 17-0175	*Pandanus* sp.	China	NA	MH412720	MH388389	[18]
*N. pernambucana*	URM7148-01 *	*Vismia guianensis*	Brazil	KJ792466	NA	KU306739	[43]
	URM7148-02	*Vismia guianensis*	Brazil	KJ792467	NA	KU306740	[43]
*N. perukae*	FMBCC 11.3 *	Guava	Pakistan	MH209077	MH460876	MH523647	[36]
*N. petila*	MFLUCC 17-1738	*Rhizophora mucronata*	Thailand	MK764275	MK764341	MK764319	[19]
	MFLUCC 17-1737 *	*Rhizophora mucronata*	Thailand	MK764276	MK764342	MK764320	[19]
*N. phangngaensis*	MFLUCC 18-0119 *	*Pandanus* sp.	Thailand	MH388354	MH412721	MH388390	[18]
*N. piceana*	CBS 254.32	*Cocos nucifera*	Indonesia	KM199372	KM199452	KM199529	[8]
	CBS 394.48 *	*Picea* sp.	UK	KM199368	KM199453	KM199527	[8]
*N. protearum*	CBS 114178 *	*Leucospermum cuneiforme* cv. “Sunbird”	Zimbabwe	JN712498	KM199463	LT853201	[44]
*N. psidii*	FMBCC 11.2 *	Guava	Pakistan	MF783082	MH477870	MH460874	[36]
*N. rhapidis*	GUCC 21501 *	*Rhododendron simsii*	China	MW931620	MW980441	MW980442	[45]
*N. rhizophorae*	MFLUCC 17-1550 *	*Rhizophora mucronata*	Thailand	MK764277	MK764343	MK764321	[19]
	MFLUCC 17-1551	*Rhizophora mucronata*	Thailand	MK764278	MK764344	MK764322	[19]
*N. rhododendri*	GUCC 21504 *	*Rhododendron simsii*	China	MW979577	MW980443	MW980444	[45]
	GUCC 21505	*Rhododendron simsii*	China	MW979576	MW980445	MW980446	[45]
*N. rosae*	CBS 101057 *	*Rosa* sp.	New Zealand	KM199359	KM199429	KM199523	[8]
	CBS 124745	*Paeonia suffruticosa*	USA	KM199360	KM199430	KM199524	[8]
*N. rosicola*	CFCC 51992 *	*Rosa chinensis*	China	KY885239	KY885245	KY885243	[15]
	CFCC 51993	*Rosa chinensis*	China	KY885240	KY885246	KY885244	[15]
*N. samarangensis*	CBS 115451	Unidentified tree	China	KM199365	KM199447	KM199556	[8]
*N. saprophytica*	MFLUCC 12-0282 *	*Magnolia* sp.	China	JX398982	JX399017	JX399048	[8]
*N. scalabiensis*	MUM 21.34 *	*Vaccinium corymbosum*	Portugal	MW969748	MW934611	MW959100	[46]
*N. sichuanensis*	CFCC 54338 *	*Castanea mollissima*	China	MW166231	MW218524	MW199750	[16]
	SM15-1C	*Castanea mollissima*	China	MW166232	MW218525	MW199751	[16]
*N. sonneratae*	MFLUCC 17-1745 *	*Sonneronata alba*	Thailand	MK764279	MK764345	MK764323	[19]
	MFLUCC 17-1744	*Sonneronata alba*	Thailand	MK764280	MK764346	MK764324	[19]
*Neopestalotiopsis sp.1*	CSUFTCC61	*Camellia oleifera*	China	OK493590	OK562365	OK507960	This study
	CSUFTCC62	*Camellia oleifera*	China	OK493591	OK562366	OK507961	This study
	CSUFTCC63	*Camellia oleifera*	China	OK493592	OK562367	OK507962	This study
*N. steyaertii*	IMI 192475 *	*Eucalyptus viminalis*	Australia	KF582796	KF582794	KF582792	[8]
*N. surinamensis*	CBS 450.74 *	Soil under *Elaeis guineensis*	Suriname	KM199351	KM199465	KM199518	[8]
*N. thailandica*	MFLUCC 17-1730 *	*Rhizophora mucronata*	Thailand	MK764281	MK764347	MK764325	[19]
	MFLUCC 17-1731	*Rhizophora mucronata*	Thailand	MK764282	MK764348	MK764326	[19]
*N. umbrinospora*	MFLUCC 12-0285 *	Unidentified plant	China	JX398984	JX399019	JX399050	[6]
*N. vaccinii*	MUM 21.36 *	*Vaccinium corymbosum*	Portugal	MW969747	MW934610	MW959099	[46]
*N. vacciniicola*	MUM 21.35 *	*Vaccinium corymbosum*	Portugal	MW969751	MW934614	MW959103	[46]
*N. vheenae*	BRIP 72293a *	*Macadamia integrifolia*	Australia	MZ303792	MZ312685	MZ344177	[21]
*N. vitis*	MFLUCC 15-1265 *	*Vitis vinifera* cv. “Summer black”	China	KU140694	KU140685	KU140676	[47]
	MFLUCC 15-1270	*Vitis vinifera* cv. “Kyoho”	China	KU140699	KU140690	KU140681	[47]
*N. zakeelii*	BRIP 72282a *	*Macadamia integrifolia*	Australia	MZ303789	MZ312682	MZ344174	[21]
*N. zimbabwana*	CBS 111495 *	*Leucospermum cunciforme*	Zimbabwe	JX556231	KM199456	KM199545	[8]
*Pestalotiopsis abietis*	CFCC 53011 *	*Abies fargesii*	China	MK397013	MK622280	MK622277	[48]
	CFCC 53012	*Abies fargesii*	China	MK397014	MK622281	MK622278	[48]
	CFCC 53013	*Abies fargesii*	China	MK397015	MK622282	MK622279	[48]
*P. adusta*	ICMP 6088 *	Refrigerator door	Fiji	JX399006	JX399037	JX399070	[6]
	MFLUCC 10-146	*Syzygium* sp.	Thailand	JX399007	JX399038	JX399071	[6]
*P. aggestorum*	LC6301 *	*Camellia sinensis*	China	KX895015	KX895348	KX895234	[12]
	LC8186	*Camellia sinensis*	China	KY464140	KY464160	KY464150	[12]
*P. anacardiacearum*	IFRDCC 2397 *	*Mangifera indica*	China	KC247154	KC247155	KC247156	[8]
*P. arceuthobii*	CBS 434.65 *	*Arceuthobium campylopodum*	USA	KM199341	KM199427	KM199516	[8]
*P. arenga*	CBS 331.92 *	*Arenga undulatifolia*	Singapore	KM199340	KM199426	KM199515	[8]
*P. australasia*	CBS 114126 *	*Knightia* sp.	New Zealand	KM199297	KM199409	KM199499	[8]
	CBS 114141	*Protea* sp.	New South Wales	KM199298	KM199410	KM199501	[8]
*P. australis*	CBS 111503	*Protea neriifolia* × *susannae* cv. “Pink Ice”	South Africa	KM199331	KM199382	KM199557	[8]
	CBS 114193 *	*Grevillea* sp.	New South Wales	KM199332	KM199383	KM199475	[8]
*P. biciliata *	CBS 124463 *	*Platanus* × *hispanica*	Slovakia	KM199308	KM199399	KM199505	[8]
	CBS 236.38	*Paeonia* sp.	Italy	KM199309	KM199401	KM199506	[8]
*P. brachiata*	LC2998 *	*Camellia* sp.	China	KX894933	KX895265	KX895150	[12]
	LC8188	*Camellia* sp.	China	KY464142	KY464162	KY464152	[12]
	LC8189	*Camellia* sp.	China	KY464143	KY464163	KY464153	[12]
*P. brassicae*	CBS 170.26 *	*Brassica napus*	New Zealand	KM199379	NA	KM199558	[8]
*P. camelliae*	MFLUCC 12-0277 *	*Camellia japonica*	China	JX399010	JX399041	JX399074	[6]
*P. camelliae-oleiferae*	CSUFTCC08 *	*Camellia oleifera*	China	OK493593	OK562368	OK507963	In this study
	CSUFTCC09	*Camellia oleifera*	China	OK493594	OK562369	OK507964	In this study
	CSUFTCC10	*Camellia oleifera*	China	OK493595	OK562370	OK507965	In this study
*P. chamaeropis*	CBS 186.71 *	*Chamaerops humilis*	Italy	KM199326	KM199391	KM199473	[6]
	LC3619	*Camellia* sp.	China	KX894991	KX895322	KX895208	[12]
*P. clavata*	MFLUCC 12-0268 *	*Buxus* sp.	China	JX398990	JX399025	JX399056	[6]
*P. colombiensis*	CBS 118553 *	*Eucalyptus eurograndis*	Colombia	KM199307	KM199421	KM199488	[8]
*P. digitalis*	MFLU 14-0208 *	*Digitalis purpurea*	New Zealand	KP781879	KP781883	NA	[49]
*P. dilucida*	LC3232 *	*Camellia sinensis*	China	KX894961	KX895293	KX895178	[12]
	LC8184	*Camellia sinensis*	China	KY464138	KY464158	KY464148	[12]
*P. diploclisiae*	CBS 115449	*Psychotria tutcheri*	China	KM199314	KM199416	KM199485	[8]
	CBS 115587 *	*Diploclisia glaucescens*	China	KM199320	KM199419	KM199486	[8]
*P. disseminata*	CBS 118552	*Eucalyptus botryoides*	New Zealand	MH553986	MH554652	MH554410	[12]
	CBS 143904	*Persea americana*	New Zealand	MH554152	MH554825	MH554587	[12]
	MEAN 1165	*Pinus pinea*	Portugal	MT374687	MT374712	MT374699	[50]
	MEAN 1166	*Pinus pinea*	Portugal	MT374688	MT374713	MT374700	[50]
*P. diversiseta*	MFLUCC 12-0287 *	*Rhododendron* sp.	China	JX399009	JX399040	JX399073	[6]
*P. doitungensis*	MFLUCC 14-0115 *	*Dendrobium* sp.	Thailand	MK993574	MK975837	MK975832	[34]
*P. dracaenicla*	MFLUCC 18-0913 *	*Dracaena* sp.	Thailand	MN962731	MN962733	MN962732	[51]
*P. dracontomelonis*	MFLU 14-0207 *	*Dracontomelon dao*	Thailand	NA	NA	KP781880	[49]
*P. ericacearum*	IFRDCC 2439 *	*Rhododendron delavayi*	China	KC537807	KC537821	KC537814	[52]
*P. etonensis*	BRIP 66615 *	*Sporobolus jacquemontii*	Australia	MK966339	MK977634	MK977635	[42]
*P. formosana*	NTUCC 17-009 *	On dead grass	China	MH809381	MH809385	MH809389	[15]
*P. furcata*	MFLUCC 12-0054 *	*Camellia sinensis*	Thailand	JQ683724	JQ683708	JQ683740	[53]
	LC6691	*Camellia sinensis*	China	KX895030	KX895363	KX895248	[12]
*P. gaultheria*	IFRD 411-014 *	*Gaultheria forrestii*	China	KC537805	KC537819	KC537812	[8]
*P. gibbosa*	NOF 3175 *	*Gaultheria shallon*	Canada	LC311589	LC311590	LC311591	[54]
*P. grevilleae*	CBS 114127 *	*Grevillea* sp.	Australia	KM199300	KM199407	KM199504	[8]
*P. hawaiiensis*	CBS 114491 *	*Leucospermum* sp.	Hawaii	KM199339	KM199428	KM199514	[8]
*P. hollandica*	CBS 265.33 *	*Sciadopitys verticillata*	Netherlands	KM199328	KM199388	KM199481	[8]
*P. hispanica*	CBS 115391 *	*Protea* cv. ‘Susara’	Spain	MH553981	MH554640	MH554399	[8]
*P. humus*	CBS 336.97 *	Soil	Papua New Guinea	KM199317	KM199420	KM199484	[8]
*P. hunanensis*	CSUFTCC15 *	*Camellia oleifera*	China	OK493599	OK562374	OK507969	In this study
	CSUFTCC18	*Camellia oleifera*	China	OK493600	OK562375	OK507970	In this study
	CSUFTCC19	*Camellia oleifera*	China	OK493601	OK562376	OK507971	In this study
*P. inflexa*	MFLUCC 12-0270 *	Unidentified tree	China	JX399008	JX399039	JX399072	[6]
*P. intermedia*	MFLUCC 12-0259 *	Unidentified tree	China	JX398993	JX399028	JX399059	[6]
*P. italiana*	MFLU 14-0214 *	*Cupressus glabra*	Italy	KP781878	KP781882	KP781881	[49]
*P. jesteri*	CBS 109350 *	*Fragraea bodenii*	Papua New Guinea	KM199380	KM199468	KM199554	[8]
*P. jiangxiensis*	LC4242	*Eurya* sp.	China	KX895035	KX895327	KX895213	[12]
	LC4399 *	*Camellia* sp.	China	KX895009	KX895341	KX895227	[12]
*P. jinchanghensis*	LC6636 *	*Camellia sinensis*	China	KX895028	KX895361	KX895247	[12]
	LC8190	*Camellia sinensis*	China	KY464144	KY464164	KY464154	[12]
*P. kandelicola*	NCYU 19-0355 *	*Kandelia candel*	China	MT560723	MT563100	MT563102	[55]
*P. kenyana*	CBS 442.67 *	*Coffea* sp.	Kenya	KM199302	KM199395	KM199502	[8]
	LC6633	*Camellia sinensis*	China	KX895027	KX895360	KX895246	[8]
*P. knightiae*	CBS 111963	*Knightia* sp.	New Zealand	KM199311	KM199406	KM199495	[8]
	CBS 114138 *	*Knightia* sp.	New Zealand	KM199310	KM199408	KM199497	[8]
*P. leucadendri*	CBS 121417 *	*Leucadendron* sp.	South Africa	MH553987	MH554654	MH554412	[56]
*P. licualacola*	HGUP 4057 *	*Licuala grandis*	China	KC492509	KC481683	KC481684	[57]
*P. linearis*	MFLUCC 12-0271 *	*Trachelospermum* sp.	China	JX398992	JX399027	JX399058	[6]
*P. longiappendiculata*	LC3013 *	*Camellia sinensis*	China	KX894939	KX895271	KX895156	[12]
*P. lushanensis*	LC4344 *	*Camellia* sp.	China	KX895005	KX895337	KX895223	[12]
	LC8182	*Camellia* sp.	China	KY464136	KY464156	KY464146	[12]
	LC8183	*Camellia* sp.	China	KY464137	KY464157	KY464147	[12]
*P. macadamiae*	BRIP 63738b *	*Macadamia integrifolia*	Australia	KX186588	KX186680	KX186621	[14]
	BRIP 63739a	*Macadamia integrifolia*	Australia	KX186589	KX186681	KX186622	[14]
	BRIP 63739b	*Macadamia integrifolia*	Australia	KX186587	KX186679	KX186620	[14]
*P. malayana*	CBS 102220 *	*Macaranga triloba*	Malaysia	KM199306	KM199411	KM199482	[8]
*P. monochaeta*	CBS 144.97 *	*Quercus robur*	Netherlands	KM199327	KM199386	KM199479	[8]
	CBS 440.83	*Taxus baccata*	Netherlands	KM199329	KM199387	KM199480	[8]
*P. nanjingensis*	CSUFTCC16 *	*Camellia oleifera*	China	OK493602	OK562377	OK507972	This study
	CSUFTCC20	*Camellia oleifera*	China	OK493603	OK562378	OK507973	This study
	CSUFTCC04	*Camellia oleifera*	China	OK493604	OK562379	OK507974	This study
*P. nanningensis*	CSUFTCC10 *	*Camellia oleifera*	China	OK493596	OK562371	OK507966	This study
	CSUFTCC11	*Camellia oleifera*	China	OK493597	OK562372	OK507967	This study
	CSUFTCC12	*Camellia oleifera*	China	OK493598	OK562373	OK507968	This study
*P. neolitseae*	NTUCC 17-011 *	On leaf of *Neolitsea villosa*	Taiwan	MH809383	MH809387	MH809391	[15]
*P. novaehollandiae*	CBS 130973 *	*Banksia grandis*	Australia	KM199337	KM199425	KM199511	[8]
*P. oryzae*	CBS 111522	*Telopea* sp.	USA	KM199294	KM199394	KM199493	[8]
	CBS 171.26	NA	Italy	KM199304	KM199397	KM199494	[8]
	CBS 353.69 *	*Oryza sativa*	Denmark	KM199299	KM199398	KM199496	[8]
*P. pandanicola*	MFLUCC 16-0255 *	*Pandanus* sp.	Thailand	MH388361	MH412723	MH388396	[18]
*P. papuana*	CBS 331.96 *	Coastal soil	Papua New Guinea	KM199321	KM199413	KM199491	[8]
	CBS 887.96	*Cocos nucifera*	Papua New Guinea	KM199318	KM199415	KM199492	[8]
*P. pallidotheae*	MAFF 240993 *	*Pieris japonica*	Japan	NR111022	LC311584	LC311585	[58]
*P. parva*	CBS 265.37 *	*Delonix regia*	NA	KM199312	KM199404	KM199508	[8]
	CBS 278.35	*Leucothoe fontanesiana*	NA	KM199313	KM199405	KM199509	[8]
*P. photinicola*	GZCC 16-0028 *	*Photinia serrulata*	China	KY092404	KY047663	KY047662	[59]
*P. portugalica*	CBS 393.48 *	NA	Portugal	KM199335	KM199422	KM199510	[8]
	LC4324	*Camellia chekiangoleosa*	China	KX895001	KX895333	KX895219	[12]
*P. pini*	MEAN 1092 *	*Pinus pinea*	Portugal	MT374680	MT374705	MT374693	[50]
*P. pinicola*	KUMCC 19-0183 *	*Pinus armandii*	China	MN412636	MN417507	MN417509	[60]
*P. rhododendri*	IFRDCC 2399 *	*Rhododendron sinogrande*	China	KC537804	KC537818	KC537811	[52]
*P. rhodomyrtus*	HGUP4230 *	*Rhodomyrtus tomentosa*	China	KF412648	KF412642	KF412645	[33]
	LC4458	*Camellia sinensis*	China	KX895010	KX895342	KX895228	[12]
*P. rhizophorae*	MFLUCC 17-0416 *	*Rhizophora apiculata*	Thailand	MK764283	MK764349	MK764327	[19]
*P. rosea*	MFLUCC 12-0258 *	*Pinus* sp.	China	JX399005	JX399036	JX399069	[6]
*P. scoparia*	CBS 176.25 *	*Chamaecyparis* sp.	NA	KM199330	KM199393	KM199478	[8]
*P. sequoiae*	MFLUCC 13-0399 *	*Sequoia sempervirens*	Italy	KX572339	NA	NA	[61]
*P. spathulata*	CBS 356.86 *	*Gevuina avellana*	Chile	KM199338	KM199423	KM199513	[8]
*P. spathuliappendiculata*	CBS 144035 *	*Phoenix canariensis*	Australia	MH554172	MH554845	MH554607	[56]
*P. telopeae*	CBS 114137	*Protea* sp.	Australia	KM199301	KM199469	KM199559	[8]
	CBS 114161 *	*Telopea* sp.	Australia	KM199296	KM199403	KM199500	[8]
	CBS 113606	*Telopea* sp.	Australia	KM199295	KM199402	KM199498	[8]
*P. terricola*	CBS 141.69 *	Soil	Pacific Islands	MH554004	MH554680	MH554438	[56]
*P. thailandica*	MFLUCC 17-1616 *	*Rhizophora apiculata*	Thailand	MK764285	MK764351	MK764329	[19]
*P. trachicarpicola*	IFRDCC 2403	*Podocarpus macrophyllus*	China	KC537809	KC537823	KC537816	[52]
	LC4523	*Camellia sinensis*	China	KX895011	KX895344	KX895230	[12]
	MFLUCC 12-0264	*Chrysophyllum* sp.	China	JX399004	JX399035	JX399068	[6]
	OP068 *	*Trachycarpus fortunei*	China	JQ845947	JQ845945	JQ845946	[62]
*P. unicolor*	MFLUCC 12-0276 *	*Rhododendron* sp.	China	JX398999	JX399030	NA	[6]
	MFLUCC 12-0275	unidentified tree	China	JX398998	JX399029	JX399063	[6]
*P. verruculosa*	MFLUCC 12-0274 *	*Rhododendron* sp.	China	JX398996	NA	JX399061	[6]
*P. yanglingensis*	LC4553 *	*Camellia sinensis*	China	KX895012	KX895345	KX895231	[12]
	LC3412	*Camellia sinensis*	China	KX894980	KX895312	KX895197	[12]
*P. yunnanensis*	HMAS 96359 *	*Podocarpus macrophyllus*	China	AY373375	NA	NA	[63]

BRIP: Queensland Plant Pathology Herbarium, Brisbane, Australia; CBS: Culture Collection of the Westerdijk Fungal Biodiversity Institute, Utrecht, The Netherlands; CFCC: China Forestry Culture Collection Center, Beijing, China; CGMCC: China General Microbiological Culture Collection Center, Institute of Microbiology, Chinese Academy of Sciences, Beijing, China; COAD: Coleção Octávio Almeida Drummond, Universidade Federal de Viçosa, Brazil; CSUFTCC: Central South University of Forestry and Technology Culture Collection, Hunan, China; FMB: Fungal Molecular Biology Laboratory, Department of Plant Pathology, University of Agriculture Faisalabad, Pakistan; GZCC: Guizhou Academy of Agricultural Sciences Culture Collection, Guizhou, China; HGUP: Plant Pathology Herbarium of Guizhou University, Guizhou, China; HMAS: Mycological Herbarium, Institute of Microbiology, Chinese Academy of Sciences, Beijing, China; ICMP: International Collection of Micro-organisms from Plants, Landcare Research, Private Bag 92170, Auckland, New Zealand; IFRDCC: International Fungal Research and Development Culture Collection; IMI: Culture Collection of CABI Europe UK Centre, Egham, UK; KNU: Kyungpook National University, Daegu, Korea; KUMCC: Kunming Institute of Botany Culture Collection, Yunnan, China; LC: working collection of Lei Cai, housed at the Institute of Microbiology, Chinese Academy of Sciences, Beijing, China; MAFF: Ministry of Agriculture, Forestry and Fisheries, Tsukuba, Ibaraki, Japan; MEAN: Instituto Nacional de Investigação Agrária e Veterinária I. P.; MFLUCC: Mae Fah Luang University Culture Collection, Chiang Rai, Thailand; MUM: Micoteca of Universidade do Minho, Portugal; NCYU: National Chiayi University, Chiayi, Taiwan; NOF: The Fungus Culture Collection of the Northern Forestry Centre, Alberta, Canada; NTUCC: the Department of Plant Pathology and Microbiology, National Taiwan University Culture Collection; URM: Culture Collection of the Universidade Federal de Pernambuco, Brazil. Ex-type strains are labeled with *. NA: Not available.

## Data Availability

All sequence data are available in NCBI GenBank following the accession numbers in the manuscript.
